# 

**Published:** 1906-04

**Authors:** 


### CONTENTS.

ORIGINAL COMMUNICATIONS—

- CAUSES OF LOSS OF CONFIDENCE IN X-RAY TREATMENT. By M. B. Hutchins, M.D., Atlanta, Ga..... 1
- IS THERE AN EPIDEMIC OF SCABIES IN THE STATE? By Bernard Wolff, M.D., Atlanta, Ga............ 4
- TRIGEMINAL NEURALGIA, SURGICAL TREATMENT. By Benjamin Merrill Ricketts, M.D., Cincinnati, O. 12
- ANOMALOUS KIDNEY. By Frank K. Boland, M.D., Atlanta, Ga.................................. 16

EDITORIAL NOTES AND COMMENTS—

- A WORD ABOUT PROPRIETARIES.................................................. 17
- THE MEDICAL ASSOCIATION OF GEORGIA.......................................... 20

NEWS AND NOTES.................................................................. 24

BOOK REVIEWS—

- A TREATISE ON SURGERY. (Fowler)............................................ 32
- CASE TEACHING IN MEDICINE. (Cabot)......................................... 33
- A REFERENCE HANDBOOK OF THE DISEASES OF CHILDREN. (Fruhwald)............... 33

CONTENTS CONTINUED.............................................................. X

